# Process Evaluation of a Rapid Evidence Support System Assessment of Ireland’s Department of Health – A Protocol

**DOI:** 10.12688/hrbopenres.14062.1

**Published:** 2025-02-03

**Authors:** Marie Tierney, Barbara Whelan, Nikita N Burke, Caitriona Creely, Trudy Duffy, Catherine Gill, Mary Horgan, John N Lavis, Teresa Maguire, Mairead O'Driscoll, John O'Neill, Elaine Toomey, Kerry Waddell, Declan Devane

**Affiliations:** 1 Evidence Synthesis Ireland, University of Galway, Galway, County Galway, Ireland; 2 School of Nursing and Midwifery, University of Galway, Galway, County Galway, Ireland; 3 Health Research Board, Dublin, Ireland; 4 Evidence for Policy Unit, Department of Further and Higher Education, Research, Innovation and Science, Dublin, Ireland; 5 Department of Health, Dublin, Ireland; 6 School of Medicine, University College Dublin, Dublin, Ireland; 7 McMaster Health Forum, McMaster University, Hamilton, Ontario, Canada; 8 Department of Health Research Methods Evidence and Impact, McMaster University, Hamilton, Ontario, Canada; 9 Health Research and innovation Policy Unit, Department of Health, Dublin, Ireland

**Keywords:** process evaluation, fidelity, acceptability, experiences, mixed methods, evidence support system, policy

## Abstract

**Background:**

The Rapid Evidence Support System Assessment (RESSA) was developed by the Global Evidence Commission to evaluate evidence support systems that inform policy decisions. These systems are designed to contextualize existing evidence, guide decision-making, and generate new insights to inform action. As evidence-informed policymaking gains traction globally, it is essential to evaluate these systems’ effectiveness. In Ireland, the Health Research Board, the Department of Health, Evidence Synthesis Ireland, Cochrane Ireland, and the Global Evidence Commission are collaborating to conduct a RESSA within the Department of Health. This process evaluation aims to assess the fidelity, acceptability, and experiences of stakeholders involved in the RESSA, providing insights for refining the methodology.

**Methods:**

The process evaluation will employ a mixed methods approach, integrating both qualitative and quantitative data collection. It will evaluate the conduct of a RESSA within the Department of Health. Fidelity assessment will examine adherence to the RESSA protocol, while acceptability will be evaluated using the Theoretical Framework of Acceptability, focusing on key stakeholders' attitudes. An exploration of the experiences of participants, capturing both facilitators and barriers to the RESSA’s success will also be conducted. Data analysis will involve thematic analysis and descriptive statistics, aiming to highlight the RESSA’s methodological strengths and areas for improvement.

**Conclusions:**

This evaluation is expected to provide critical insights into the strengths and limitations of the RESSA methodology, with implications for evidence-informed policymaking. Findings will offer recommendations to enhance the robustness and applicability of the RESSA in Ireland and beyond. Dissemination will include academic publications and reports, contributing to the broader understanding of effective evidence support systems. This process evaluation aims to inform future RESSAs and strengthen the evidence support framework, ensuring better-informed policy decisions at local, national, and international levels.

## Introduction

The evidence support system in a given jurisdiction is a set of structures or processes focused on contextualising existing evidence for advisory and decision-making processes and for learning and improvement platforms, and on building new evidence to inform future decision-making ( Global Commission on Evidence to Address Societal Challenges, 2024). Evidence support systems play a crucial role in informing policy decisions across various sectors. These systems encompass structures and processes that contextualise existing evidence, support learning and improvement and generate new insights to guide future decision-making.

As governments worldwide increasingly recognise the value of evidence-informed policymaking, there is a growing need to assess and enhance the effectiveness of these support systems ( Parkhurst, 2016). Integrating evidence into policy processes presents significant challenges. Cairney and Oliver (2017) detail the importance of understanding the political dimensions of evidence use, arguing that policymaking is influenced by various competing factors. Boaz *et al.*, 2011 examines the practical challenges of implementing evidence in policy contexts, highlighting the need for effective mechanisms to support the translation of evidence into practice. Similarly, Oliver *et al.*, 2014 have identified key barriers and facilitators to the use of evidence by policymakers, underscoring the importance of robust evidence support systems.

Despite these insights, there remains a need for further exploration of how evidence support systems operate in different contexts, the challenges they face, and the strategies that can enhance their effectiveness.

In Ireland, the focus on evidence-informed policymaking has intensified, particularly in the wake of the COVID-19 pandemic ( Clyne *et al.*, 2023). This has led to a collaboration between the Health Research Board (HRB), the Department of Health, Evidence Synthesis Ireland (ESI) and Cochrane Ireland, and the Global Commission on Evidence to Address Societal Challenges (Global Evidence Commission). The primary aim of this collaboration is to assess and strengthen the evidence support system within the Department of Health in Ireland using the Rapid Evidence Support System Assessment (RESSA) process.

The RESSA process, developed by the Global Evidence Commission, provides a structured approach to evaluating evidence support systems in various countries. The RESSA process involves analysing websites and documents and conducting interviews with key personnel to gather information about the features of evidence support systems and inform action based on the findings ( Global Commission on Evidence to Address Societal Challenges, 2024). Currently, RESSAs are being conducted in 12 countries worldwide ( Global Commission on Evidence to Address Societal Challenges, 2024), including Ireland. The RESSA in Ireland aims to comprehensively evaluate Ireland's evidence-support ecosystem for health policymaking and seeks to provide insights for the Department of Health, inform the HRB's strategic decisions, and develop robust methodologies applicable across various sectors.

In this RESSA, a flexible, mixed methods approach, integrating qualitative and quantitative analyses will be adopted. It will be conducted in four main stages: 1. high-level website review; 2. in-depth website and document review; 3. key informant interviews; 4. seek feedback. The high-level website review will involve a systematic examination of relevant websites to identify key stakeholders, evidence-related activities, and publicly available resources within the evidence-support system. The in-depth website and document review will entail a more detailed analysis of the Department of Health website to gather information on how evidence support is approached from the evidence-demand side, evidence-supply side and at the interface between the two. The key informant interviews will involve in-depth, semi-structured interviews with approx. 10–20 key informants strategically selected through purposive and respondent-driven sampling to represent a diverse range of perspectives within the health policy-making evidence ecosystem, including policymakers and researchers from the Department of Health and other key stakeholders such as the Health Information and Quality Authority (HIQA), Economic and Social Research Institute (ESRI) and HRB. The seeking feedback stage will involve provision of feedback on the main findings of the RESSA by the oversight group. In addition, champions identified through the RESSA process and interviewed will be contacted by e-mail for their feedback.

Given the concurrent implementation of RESSAs in multiple countries and the likelihood of future evaluations, it is essential to learn from the current processes to enhance the efficiency and effectiveness of future RESSAs. To achieve this, a process evaluation will be employed to assess the implementation and identify potential improvements. The purpose of this process evaluation is to determine the strengths and weaknesses of the methodology used in the jurisdictional assessment, with recommendations for future applications of the methodology.

Typically, a process evaluation is a method for assessing the implementation and potential improvements of a process. It involves collecting, analysing and using data to determine the effectiveness of a process and identify its strengths and weaknesses. Process evaluation is systematic and inductive, aiming to maximise learnings and improve programmes.

Guidance for conducting process evaluations is well-established across various research domains, providing essential frameworks and methodologies to ensure that evaluations are systematic and effective. The Medical Research Council (MRC) offers comprehensive guidelines specifically designed for process evaluations of complex interventions. These guidelines emphasise the importance of understanding how interventions are implemented, exploring the mechanisms through which they produce effects, and considering the contextual factors that influence outcomes ( Moore *et al.*, 2015). This approach ensures that evaluators can systematically assess the fidelity and adaptation of interventions in real-world settings. In addition to the MRC guidelines, the framework provided by Hulscher *et al.* (2003) for quality improvement interventions is another valuable resource. This framework focuses on the detailed assessment of implementation processes in healthcare settings, helping to identify barriers and facilitators to successful interventions. Furthermore, the Consolidated Framework for Implementation Research (CFIR) offers a structured approach to evaluating the implementation of interventions, particularly within healthcare contexts ( Damschroder *et al.*, 2009). The CFIR is widely used to guide the systematic examination of factors that affect implementation outcomes, making it a critical tool for process evaluations.

To our knowledge, no framework exists for conducting a process evaluation of an assessment like a RESSA. Drawing from these best practice frameworks, this process evaluation of a RESSA conducted at Ireland’s Department of Health will examine the fidelity, acceptability, and experiences of stakeholders involved in the RESSA process.

## Aims and objectives

The aim of this study is to conduct a process evaluation to assess the methodological process of the RESSA, emphasising successes, challenges, and recommendations for future applications of the methodology.

To achieve this aim, the objectives are:

1) Assess the fidelity of the RESSA to evaluate whether it was conducted as planned and identify areas for improvement in its conduct

2) Assess the acceptability of the RESSA to key informants, researchers, and the oversight group, focusing on their perceptions and attitudes towards the assessment process

3) Explore the experiences of key stakeholders involved in the RESSA to understand the facilitators and barriers to its success and gather insights for refining conduct of future RESSAs.

## Methods

### Study design

A concurrent mixed methods design will be employed to integrate qualitative and quantitative findings. This approach ensures triangulation of data sources and methodologies, enhancing the robustness and reliability of the evaluation. The process evaluation focuses on three key aspects: fidelity, acceptability, and stakeholder experiences. By combining quantitative and qualitative methods, we aim to gain a holistic understanding of the RESSA process and identify areas for improvement in future applications. The evaluation will run in tandem with the RESSA.

### Study population

The study will involve three participant groups:

1. RESSA key informants: Policymakers, researchers and stakeholders from the Department of Health, HRB, and affiliated organisations such as HIQA and ESRI. Participants will be purposively sampled to ensure diverse representation within the health policymaking ecosystem.
2. RESSA oversight group: Members from the Global Evidence Commission, Department of Health, Department of Further and Higher Education, Research, Innovation and Science (Evidence for Policy Unit) and ESI/Cochrane Ireland.
3. RESSA Researcher: The individual responsible for conducting the RESSA, who is an experienced researcher at ESI at the University of Galway,

### Outcome measures

***Fidelity.*** Fidelity refers to the extent to which an intervention is conducted as planned ( Durlak & DuPre, 2008). It is a multifaceted construct that requires the assessment of multiple domains. ( Feely *et al.*, 2018) recommend that the scope of a fidelity assessment should match the needs of the study and provide key information for future research projects.

Assessing fidelity accurately, combined with a high degree of adherence to the intervention, is critical to the reliability, validity, replicability, and scalability of the results of an intervention research study Feely *et al.* (2018). When an intervention is delivered as per protocol and a high level of fidelity is achieved, researchers can have greater confidence in the research outcomes ( Moore *et al.*, 2015). Understanding and measuring whether an assessment like a RESSA has been conducted with fidelity is essential for researchers and practitioners to gain insights into how and why an assessment works or does not work and to what extent outcomes can be improved. Assessing fidelity at multiple levels helps guide efforts to improve fidelity, thereby enhancing the internal and external validity of future iterations of the intervention.

In this study, the following constructs of fidelity are pertinent to assess:

- Fidelity of training: assessing if all individuals involved in the conduct of the RESSA are adequately trained and that the training is delivered as intended.
- Fidelity of delivery: assessing if the assessment is conducted (i.e. delivered) according to the protocol, including adherence to the planned procedures and content of the RESSA.

These were chosen as they represent critical aspects of the RESSA process that could significantly impact its outcomes. By focusing on these constructs, we aim to identify areas where fidelity may be compromised and take steps to address these issues in future assessments.

To establish the self-competence of the researcher conducting the RESSA, and to explore any barriers or scope for improvement with regard to training, an interview guide (Appendix 1) was developed by the research team and oversight group. A semi-structured interview will be conducted by the lead author with the RESSA researcher subsequent to the completion of the RESSA. The interview will be audio-recorded and transcribed verbatim. We will conduct a thematic analysis of the interview data using a framework informed by the study's interview guide. The interview guide, developed based on the study aims, will inform the initial coding framework. A coding framework will be developed iteratively, with an initial set of codes derived directly from the interview questions and prompts. During the analysis, inductive coding will also be applied to identify themes that extend beyond the predefined framework, capturing unanticipated insights and participant experiences. Coding will be facilitated using NVivo V20 ( QSR International, 2020).

To assess whether the RESSA was delivered as designed, a mixed methods approach will be utilised. To assess whether the RESSA components show fidelity to the intended process, we will quantitatively compare the conduct of the RESSA website and document searches and RESSA interviews against the RESSA protocol. This comparison will be performed using a detailed checklist (Appendix 2) that outlines each component of the RESSA protocol. Two members of the RESSA research team will independently score this checklist to determine whether each component was delivered as intended during the delivery of the RESSA process. The checklist items will include specific criteria related to the website and document searches as well as the conduct of the interviews. Each item on the checklist will be scored as "met" or "not met" based on the adherence to the protocol. After both researchers have independently scored the checklist, their scores will be compared. Any discrepancies between the two sets of scores will be discussed in detail to understand the reasons for the differences. Through discussion, the researchers will aim to reach a consensus on each item. If consensus cannot be reached, a third researcher will be consulted to make the final decision. To quantify the level of agreement between the conduct of the RESSA and the protocol, we will calculate the percentage agreement. The percentage agreement will be determined using the following formula:


Percentageagreement=(Numberofagreed"metitems"/Totalnumberofitems)×100

Here, Number of agreed "met items" refers to the number of checklist items where both researchers independently scored the item as "met". "Total Number of Items" refers to the total number of checklist items assessed. This percentage agreement will be reported to indicate the level of consistency between the conduct of the RESSA and the protocol. A high percentage agreement will suggest strong fidelity to the RESSA protocol, while a lower percentage may indicate areas where the conduct diverged from the intended protocol. Additionally, the nature and reasons for any discrepancies will be documented and discussed to provide further insights into the fidelity assessment process.

We will also conduct a quantitative assessment to determine the number of key informants who:

- Declined interview
- Delegated interview
- Did not attend the interview
- Did not respond to the interview request
- Refused to answer question(s) during the interview

Descriptive statistics will be used to summarise the quantitative data. Specific statistical methods, such as frequency distributions, means, and standard deviations, will be applied to interpret the data. Benchmarks or thresholds for evaluation will be established based on relevant literature and preliminary data.

Barriers and facilitators: The research team and oversight group have developed an interview guide (Appendix 2) to explore any barriers or scope for improvement with regard to the delivery of the RESSA. After the completion of all components of the RESSA, a semi-structured interview will be conducted with the RESSA researcher using this interview guide. The interviews will be audio-recorded and transcribed verbatim. We will use thematic analysis to explore the interview data, guided by a coding framework derived from the interview guide. The interview guide, designed to reflect the study aims, will shape the initial set of deductive codes. This structured approach will ensure that the analysis remains aligned with the study’s objectives and the key areas of inquiry. To complement this deductive approach, we will apply inductive coding to capture unexpected insights and themes emerging organically from the data. NVivo V20 ( QSR International, 2020) will be used to facilitate the management and analysis of the data.

***Acceptability.*** In healthcare interventions, acceptability is critical for both successful implementation and the evaluation of outcomes ( Sekhon *et al.*, 2017). Traditionally perceived as a singular attribute, recent research suggests that acceptability is inherently multi-faceted, involving various dimensions that influence both the delivery and reception of assessments. As noted by Diepeveen *et al.* (2013), the acceptability of an intervention to both deliverers and recipients is crucial for its efficacy and adherence to protocol.

Acceptability significantly influences user engagement, adherence to assessment protocols, and ultimately, the success of the assessment itself. According to ( Fisher *et al.*, 2006) and ( Hommel *et al.*, 2013), when participants find an intervention acceptable, they are more likely to adhere to its recommendations and experience beneficial outcomes. This connection underscores the necessity of incorporating a robust assessment of acceptability into process evaluations to understand the factors influencing the RESSA’s effectiveness.

The MRC has highlighted the increasing importance of acceptability in its published guidance documents for designing and evaluating complex interventions ( Moore *et al.*, 2015; Skivington *et al.*, 2021). These documents show a growing recognition of acceptability, with each new release including more references to this construct, signifying its expanding role in process evaluations. This trend underscores the MRC's commitment to ensuring that process evaluations adequately consider how acceptability impacts the success of assessments.

The Theoretical Framework of Acceptability (TFA), developed by Sekhon *et al.* (2017), provides a structured approach to evaluate acceptability. This framework identifies seven critical components: affective attitude, burden, ethicality, intervention coherence, opportunity costs, perceived effectiveness, and self-efficacy. Each component offers a distinct perspective on how assessments are received by participants, allowing for a comprehensive evaluation of their responses, ranging from cognitive and emotional reactions to perceived burdens and benefits. For this RESSA process evaluation, the TFA will be used due to its evidence-based, multidimensional construct, which aligns well with our aim to thoroughly understand and document users’ feedback to the RESSA. The framework facilitates a detailed assessment of each aspect of acceptability and enhances the interpretation of how these factors may affect overall engagement and effectiveness. By evaluating acceptability through the lens of the TFA, we aim to gain deeper insights into the factors that facilitate or hinder the success of this RESSA, thereby contributing to more effective strategies in future RESSAs.

To thoroughly assess acceptability, we will recruit ten participants who participated in the RESSA, based on the work of ( Francis *et al.* (2010), which recommends collecting data from an initial sample of ten participants and then applying a stopping criterion of an additional three participants until saturation is reached for theory-based interview studies. Semi-structured interview guides (Appendices 3, 4), grounded in the seven constructs of the TFA, have been developed to retrospectively examine the acceptability of the RESSA across the three participant groups. Interviews will be audio-recorded and transcribed verbatim to ensure an accurate and detailed dataset for analysis. The analysis will adopt a deductive thematic approach, utilising the TFA as the coding framework to systematically explore participants' experiences and perceptions of the RESSA process. NVivo V20 software ( QSR International, 2020) will be employed to manage, organise, and facilitate the coding and analysis of the qualitative data. By applying the TFA within the context of RESSA, we aim to analyse how each of its seven constructs relates to participants’ interactions with and perspectives on the RESSA methodology. This comprehensive approach will provide a nuanced assessment of the acceptability of RESSA, offering valuable insights to inform and refine future applications.

***Experiences.*** To improve engagement with future RESSAs, it is essential to thoroughly understand the experiences of participants within the current assessment and to identify any barriers and facilitators to its success. A comprehensive exploration of participant experiences will yield vital insights that can inform the development and refinement of future iterations.

The objective of this component of the process evaluation is to delineate and examine the facilitators and barriers to the RESSA process as perceived by key stakeholders. These stakeholders include RESSA key informants, researchers, and members of the oversight group. By engaging with these perspectives, the evaluation seeks to capture a holistic view of the assessment's operational dynamics.

It is important to gain insights from those directly involved in the RESSA process. Understanding these perspectives will not only help identify factors that either support or hinder the assessment but also ensure that the RESSA is effectively tailored to meet the needs and preferences of its users. Such an approach is crucial for refining the assessment for future rollouts, whether in Ireland or other jurisdictions.

The insights gathered from this evaluation will be important in refining the RESSA and enhancing its relevance, utility, and effectiveness. By integrating stakeholder feedback into the development process, the RESSA can be strategically adapted to be more responsive to the specific needs and preferences of the key stakeholders.

The research team and oversight group developed interview guides (Appendices 5, 6, 7) to understand the usefulness of the RESSA and its outputs to the Department of Health, as well as barriers to and facilitators of its success for each participant group. The assessment of usefulness will include questions about the relevance, applicability, and potential impact of RESSA findings on the evidence support system within the Department of Health. Semi-structured interviews will be conducted retrospectively with the three RESSA participant groups. We will seek to interview 50% of the RESSA key informants and oversight group. Interviews will be audio-recorded and transcribed verbatim. We will conduct a thematic analysis of the interview data, guided by a coding framework informed by the study’s interview guide. The interview guide, developed by the authors to align with the aims of the study, will provide the foundation for an initial set of deductive codes. This structured approach will ensure that the analysis remains focused on the study’s objectives and primary areas of interest. The analysis will be facilitated using NVivo V20 ( QSR International, 2020) to maintain consistency and credibility throughout the process.

### Trustworthiness

Trustworthiness has been a consistent concern with qualitative research ( Nowell *et al.*, 2017). This protocol has made several provisions to address Guba’s criteria for trustworthiness ( Guba, 1981) in qualitative research to validate the findings.

We will adopt appropriate, well-recognised research methods, utilise random sampling of individuals serving as informants and utilise triangulation via the use of different methods and different types of informants. We will use iterative questioning in data collection dialogues and allow for peer scrutiny of the project. We will also provide an in-depth methodological description with detailed records of data collection and analysis procedures to provide an audit trail and allow for the study to be repeated and also to allow the integrity of the results to be scrutinised.

### Ethical considerations

An application for the RESSA process evaluation was approved by the University of Galway Research Ethics Committee (Ref: 2024.01.003, Amend 2024.03).

Informed consent will be obtained from all participants before data collection. Participants will be provided with detailed information about the study, including its purpose, procedures, potential risks, and benefits. Confidentiality will be ensured by assigning unique identifiers to participants and securely storing consent forms. Measures to protect sensitive information and handle any potential risks to participants will be detailed in the consent forms.

### Data management and privacy

All audio recordings from interviews will be deleted after transcription. Only anonymised transcripts will be retained. These transcripts, along with any field notes and related documents, will be stored for a minimum of seven years in accordance with University of Galway policy.

In compliance with GDPR 2018 and the University of Galway Personal Data Security Schedule (PDSS), electronic records will be stored on the University of Galway OneDrive server. Access to these records will be through password-protected, encrypted laptops or desktops belonging to the research team. Consent forms will be securely stored either electronically in OneDrive or physically in a locked filing cabinet at the University of Galway. Individual names will not be linked to responses at any stage of the study.

Access to the data is restricted to the research team members: Dr. Marie Tierney, Professor Declan Devane, Dr. Nikita Burke, and Dr. Barbara Whelan. If a professional transcription service is utilised, it will be bound by stringent data confidentiality agreements.

### Dissemination

The dissemination of the work of this process evaluation will be multi-faceted.

We will publish this protocol of a process evaluation of a RESSA in a peer-reviewed journal. As previously noted, there has not been a framework for the conduct of a process evaluation of an assessment like a RESSA previously, so this protocol will serve to guide future process evaluations of RESSAs.

We will also publish the findings of the RESSA process evaluation and produce a report for our funders, the HRB. We anticipate that the findings of the RESSA process evaluation will offer insights into its effectiveness and help inform the methodology that could be applied across governmental departments, ultimately enhancing evidence-informed policymaking across the board.

Furthermore, we will present both the protocol and the findings of the process evaluation at the “RESSA Country Leads” meetings led by the Global Evidence Commission to provide insights to others working in this space internationally.

## Conclusion

This protocol outlines a structured evaluation of the Rapid Evidence Support System Assessment (RESSA) within Ireland’s Department of Health. By focusing on fidelity, acceptability, and stakeholder experiences, this study aims to enhance the RESSA methodology's relevance and impact. The findings will contribute to refining how evidence support systems operate, improving their adaptability and robustness in diverse policy contexts.

As one of the first systematic evaluations of the RESSA, this study has the potential to inform evidence-informed policymaking both in Ireland and internationally. The results will offer insights into effective approaches for integrating evidence into policy development and decision-making processes, fostering a more consistent and context-sensitive application of evidence-informed policy. Ultimately, this protocol seeks to support the evolution of evidence support systems, enhancing their capacity to guide strategic decisions in government and beyond.

## Ethical considerations and consent

An application for the RESSA process evaluation was approved by the University of Galway Research Ethics Committee (Ref: 2024.01.003, Amend 2024.03) on 26-03-2024. Participant information sheets and consent forms were approved during the University of Galway ethics approval process. Written informed consent will be obtained from all participants before data collection. Participants will be provided with detailed information about the study, including its purpose, procedures, potential risks, and benefits. Confidentiality will be ensured by assigning unique identifiers to participants and securely storing consent forms. The study will be performed according to the Declaration of Helsinki.

## Data Availability

No data are associated with this article. Open Science Framework: Process Evaluation of a Rapid Evidence Support System Assessment of Ireland’s Department of Health – A Protocol, https://doi.org/10.17605/OSF.IO/W5QAN ( Tierney, 2024). Extended data in this project pertains to interview guides and data collection tools to measure the constructs of fidelity, acceptability and experiences among the three participant groups. It also pertains to the COREQ checklist. This project contains the following referenced extended data: Appendix 1: Fidelity of training – RESSA researcher Appendix 2: Fidelity of delivery – RESSA researcher Appendix 3: Acceptability - RESSA key informants Appendix 4: Acceptability – Oversight group Appendix 5: Experiences – RESSA key informants Appendix 6: Experiences – RESSA researcher Appendix 7: Experiences – Oversight group Appendix 8: COREQ checklist Data are available under the terms of the Creative Commons Attribution 4.0 International license (CC-BY 4.0). We will use the COnsolidated criteria for REporting Qualitative research (COREQ) checklist (Appendix 8), as developed by Tong *et al.* in 2007, when reporting our findings. The use of this checklist promotes explicit and comprehensive reporting of qualitative research and ensures that researchers provide sufficient detail on their methods of data analysis and the relationship between the analysis and the findings in the research report so that reviewers can assess the rigour of the analysis and the credibility of the findings.
