# Process Evaluation of a Rapid Evidence Support System Assessment of Ireland’s Department of Health – A Protocol

**DOI:** 10.12688/hrbopenres.14062.2

**Published:** 2025-10-13

**Authors:** Marie Tierney, Barbara Whelan, Nikita N Burke, Caitriona Creely, Trudy Duffy, Catherine Gill, Mary Horgan, John N Lavis, Teresa Maguire, Mairead O'Driscoll, John O'Neill, Elaine Toomey, Kerry Waddell, Declan Devane

**Affiliations:** 1 Evidence Synthesis Ireland, University of Galway, Galway, County Galway, Ireland; 2 School of Nursing and Midwifery, University of Galway, Galway, County Galway, Ireland; 3 Health Research Board, Dublin, Ireland; 4 Evidence for Policy Unit, Department of Further and Higher Education, Research, Innovation and Science, Dublin, Ireland; 5 Department of Health, Dublin, Ireland; 6 School of Medicine, University College Dublin, Dublin, Ireland; 7 McMaster Health Forum, McMaster University, Hamilton, Ontario, Canada; 8 Department of Health Research Methods Evidence and Impact, McMaster University, Hamilton, Ontario, Canada; 9 Health Research and Innovation Policy Unit, Department of Health, Dublin, Ireland

**Keywords:** process evaluation, fidelity, acceptability, experiences, mixed methods, evidence support system, policy

## Abstract

**Background:**

The Rapid Evidence Support System Assessment (RESSA) was developed by the Global Evidence Commission to evaluate evidence support systems that inform policy decisions. These systems are designed to contextualise existing evidence, guide decision-making, and generate new insights to inform action. As evidence-informed policymaking gains traction globally, it is essential to evaluate these systems’ effectiveness. In Ireland, the Health Research Board, the Department of Health, Evidence Synthesis Ireland, Cochrane Ireland, and the Global Evidence Commission are collaborating to conduct a RESSA within the Department of Health. This process evaluation aims to assess the fidelity, acceptability, and experiences of stakeholders involved in the RESSA, providing insights for refining the methodology.

**Methods:**

The process evaluation will employ a mixed methods approach, integrating both qualitative and quantitative data collection. It will evaluate the conduct of a RESSA within the Department of Health. Fidelity assessment will examine adherence to the RESSA protocol, while acceptability will be evaluated using the Theoretical Framework of Acceptability, focusing on key stakeholders' attitudes. An exploration of the experiences of participants, capturing both facilitators and barriers to the RESSA’s success will also be conducted. Data analysis will involve thematic analysis and descriptive statistics, aiming to highlight the RESSA’s methodological strengths and areas for improvement.

**Conclusions:**

This evaluation is expected to provide critical insights into the strengths and limitations of the RESSA methodology, with implications for evidence-informed policymaking. Findings will offer recommendations to enhance the robustness and applicability of the RESSA in Ireland and beyond. Dissemination will include academic publications and reports, contributing to the broader understanding of effective evidence support systems. This process evaluation aims to inform future RESSAs and strengthen the evidence support framework, ensuring better-informed policy decisions at local, national, and international levels.

## Introduction

The evidence support system in a given jurisdiction is a set of structures or processes focused on contextualising existing evidence for advisory and decision-making processes and for learning and improvement platforms, and on building new evidence to inform future decision-making ( Global Commission on Evidence to Address Societal Challenges, 2024). Evidence support systems play a crucial role in informing policy decisions across various sectors. These systems encompass structures and processes that contextualise existing evidence, support learning and improvement and generate new insights to guide future decision-making.

As governments worldwide increasingly recognise the value of evidence-informed policymaking, there is a growing need to assess and enhance the effectiveness of these support systems ( Parkhurst, 2016). Integrating evidence into policy processes presents significant challenges. Cairney and Oliver (2017) detail the importance of understanding the political dimensions of evidence use, arguing that policymaking is influenced by various competing factors. Boaz *et al.*, 2011 examines the practical challenges of implementing evidence in policy contexts, highlighting the need for effective mechanisms to support the translation of evidence into practice. Similarly, Oliver *et al.*, 2014 have identified key barriers and facilitators to the use of evidence by policymakers, underscoring the importance of robust evidence support systems.

Despite these insights, there remains a need for further exploration of how evidence support systems operate in different contexts, the challenges they face, and the strategies that can enhance their effectiveness.

In Ireland, the focus on evidence-informed policymaking has intensified, particularly in the wake of the COVID-19 pandemic ( Clyne *et al.*, 2023). This has led to a collaboration between the Health Research Board (HRB), the Department of Health, Evidence Synthesis Ireland (ESI) and Cochrane Ireland, and the Global Commission on Evidence to Address Societal Challenges (Global Evidence Commission). The primary aim of this collaboration is to assess and strengthen the evidence support system within the Department of Health in Ireland using the Rapid Evidence Support System Assessment (RESSA) process ( Whelan *et al*., 2025).

To support learning, the conduct of the RESSA will be accompanied by a process evaluation to examine its fidelity, acceptability, and stakeholder experiences. The process evaluation aims to identify successes, challenges, and recommendations to strengthen future RESSAs. Given the concurrent implementation of RESSAs in multiple countries and the likelihood of future evaluations, it is essential to learn from the current processes to enhance the efficiency and effectiveness of future RESSAs. To achieve this, a process evaluation will be conducted to assess the implementation and identify potential areas for improvement. The purpose of this process evaluation is to determine the strengths and weaknesses of the methodology used in the jurisdictional assessment, with recommendations for future applications of the methodology.

Typically, a process evaluation is a method for assessing the implementation and potential improvements of a process. It involves collecting, analysing and using data to determine the effectiveness of a process and identify its strengths and weaknesses. Process evaluation is systematic and inductive, aiming to maximise learning and improve programmes.

Guidance for conducting process evaluations is well-established across various research domains, providing essential frameworks and methodologies to ensure that evaluations are systematic and effective. The Medical Research Council (MRC) provides comprehensive guidelines specifically designed for evaluating the processes of complex interventions. These guidelines emphasise the importance of understanding how interventions are implemented, exploring the mechanisms through which they produce effects, and considering the contextual factors that influence outcomes ( Moore *et al.*, 2015). This approach ensures that evaluators can systematically assess the fidelity and adaptation of interventions in real-world settings. In addition to the MRC guidelines, the framework provided by Hulscher *et al.* (2003) for quality improvement interventions is another valuable resource. This framework focuses on the detailed assessment of implementation processes in healthcare settings, helping to identify barriers and facilitators to successful interventions. Furthermore, the Consolidated Framework for Implementation Research (CFIR) provides a structured approach to evaluating the implementation of interventions, particularly within healthcare contexts ( Damschroder *et al.*, 2009). The CFIR is widely used to guide the systematic examination of factors that affect implementation outcomes, making it a critical tool for process evaluations.

To our knowledge, no framework exists for conducting a process evaluation of an assessment like a RESSA. Drawing from these best practice frameworks, this process evaluation of a RESSA conducted at Ireland’s Department of Health will examine the fidelity, acceptability, and experiences of stakeholders involved in the RESSA process.

## Aims and objectives

The aim of this study is to conduct a process evaluation to assess the methodological process of the RESSA, emphasising successes, challenges, and recommendations for future applications of the methodology.

To achieve this aim, the objectives are:

1) Assess the fidelity of the RESSA to evaluate whether it was conducted as planned and identify areas for improvement in its conduct

2) Assess the acceptability of the RESSA to key informants, researchers, and the oversight group, focusing on their perceptions and attitudes towards the assessment process

3) Explore the experiences of key stakeholders involved in the RESSA to understand the facilitators and barriers to its success and gather insights for refining conduct of future RESSAs.

## Methods

### Study design

**Figure 1.**
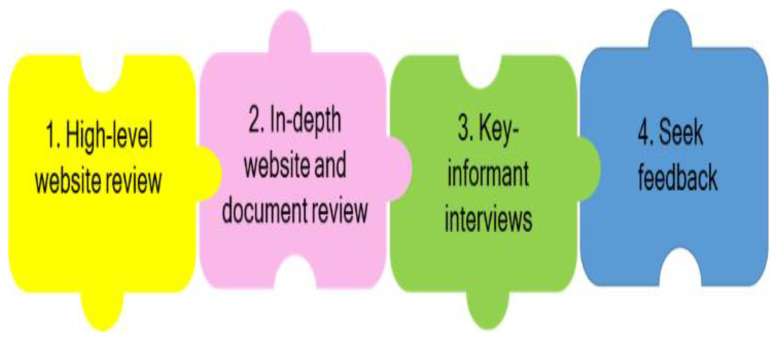
Overview of the four stages of the RESSA.

This process evaluation will be conducted in parallel with the RESSA being undertaken within the Department of Health. The RESSA is described in detail elsewhere ( Whelan *et al*., 2025) and uses a structured, mixed-methods approach to assess the evidence-support ecosystem for health policymaking. In brief, the RESSA involves four main stages: (i) a high-level website review to identify stakeholders and evidence-related activities; (ii) an in-depth website and document review to analyse evidence-demand and evidence-supply mechanisms; (iii) semi-structured key informant interviews with approximately 10–20 stakeholders; and (iv) feedback on the main findings from the oversight group and other champions identified during the process ( Figure 1).

A concurrent mixed methods design will be employed to integrate qualitative and quantitative findings. This approach ensures triangulation of data sources and methodologies, enhancing the robustness and reliability of the evaluation. The process evaluation focuses on three key aspects: fidelity, acceptability, and stakeholder experiences. By combining quantitative and qualitative methods, we aim to gain a holistic understanding of the RESSA process and identify areas for improvement in future applications. The evaluation will run in tandem with the RESSA.

### Study population and recruitment

The study will involve three participant groups:

1. RESSA key informants: Policymakers, researchers and stakeholders from the Department of Health, HRB, and affiliated organisations such as HIQA and ESRI. Participants will be purposively sampled to ensure diverse representation within the health policymaking ecosystem. Approximately 10–20 key informants will be interviewed as part of the RESSA, and the process evaluation aims to include at least 50% of these participants, purposively sampled to maximise variation in perspectives.
2. RESSA oversight group: Members from the Global Evidence Commission, Department of Health, Department of Further and Higher Education, Research, Innovation and Science (Evidence for Policy Unit) and ESI/Cochrane Ireland. At least 50% of oversight group members (anticipated n≈3–4) will be invited to participate in the process evaluation to ensure diversity of views while minimising respondent burden.
3. RESSA Researcher: The individual responsible for conducting the RESSA, who is an experienced researcher at ESI at the University of Galway, will be interviewed to provide a detailed account of training, delivery, and perceived barriers and enablers.

Recruitment will use purposive sampling with respondent-driven referral, where needed, to ensure diverse perspectives. Invitations will be sent by email with participant information sheets and consent forms, and follow-up reminders will be issued to those who do not initially respond.

### Outcome measures

***Fidelity.*** Fidelity refers to the extent to which an intervention is conducted as planned ( Durlak & DuPre, 2008). It is a multifaceted construct, and accurate assessment is critical to ensure the reliability, validity, replicability, and scalability of intervention research ( Feely *et al*. (2018); Moore *et al*., 2015). Understanding and measuring whether an assessment like a RESSA has been conducted with fidelity is essential for researchers and practitioners to gain insights into how and why an assessment works or does not work and informs future iterations of the intervention.

In this study, the following constructs of fidelity are pertinent to assess:

- Fidelity of training: assessing if all individuals involved in the conduct of the RESSA are adequately trained and that the training is delivered as intended.
- Fidelity of delivery: assessing if the assessment is conducted (i.e. delivered) according to the protocol, including adherence to the planned procedures and content of the RESSA.

This data will be used to identify areas where fidelity may be compromised and take steps to address these issues in future assessments.

***Acceptability.*** Acceptability reflects the extent to which participants and deliverers perceive an intervention as appropriate, relevant, and feasible, based on their cognitive and emotional responses ( Sekhon *et al*., 2017). It is a critical determinant of implementation success and has been highlighted as a key construct in MRC guidance on the evaluation of complex intervention processes ( Skivington *et al*., 2021).

This evaluation will examine acceptability across the seven domains of the Theoretical Framework of Acceptability (TFA) ( Sekhon *et al*., 2017):

- *Affective attitude:* Participants’ feelings about taking part in the RESSA
- *Burden:* The perceived amount of effort required to participate
- *Ethicality:* Whether participation was consistent with stakeholders’ values
- *Coherence:* The extent to which participants understood the purpose and process of the RESSA
- *Opportunity costs:* Perceptions of what participants had to give up (e.g. time, other priorities)
- *Perceived effectiveness:* Whether participants believed the RESSA could achieve its stated goals
- *Self-efficacy:* Confidence in their ability to engage with or contribute to the RESSA

Assessing these domains will provide a comprehensive understanding of how the RESSA was delivered and received by stakeholders and highlight areas that could improve future uptake and engagement.

***Experiences.*** Stakeholder experience is an important construct to complement fidelity and acceptability, capturing contextual factors that influence whether the RESSA is useful and feasible.

This aspect of the evaluation will explore:

- Perceived usefulness and relevance: How RESSA findings were perceived in terms of supporting decision-making or contributing to the evidence support system
- Timeliness: Whether stakeholders considered the RESSA outputs to have been delivered at a useful time for decision-making
- Success elements: Features that contributed to a smooth delivery
- Barriers and challenges: Factors that hindered the process
- Lessons learned and recommendations: Stakeholders’ suggestions for improving future RESSAs.

These outcomes will help identify practical steps to strengthen future iterations of the RESSA and ensure that it can effectively meet the needs of decision-makers.

### Data collection procedures

- Fidelity Checklist
  The published RESSA protocol ( Whelan *et al*., 2025) was adapted into a structured checklist covering each step of the four RESSA stages. Each step will be scored as “met” or “not met,” with space to record notes on adaptations or deviations where applicable.
- Fidelity Process Data
  Quantitative fidelity indicators will include counts of key informants who declined, delegated, or did not attend their interview; those who did not respond to the interview request; and those who refused to answer one or more questions during the interview. These will be summarised using descriptive statistics (frequencies, means, standard deviations) to contextualise participation and feasibility.
- Semi-Structured Interviews
  Semi-structured interviews will be conducted retrospectively with the individuals representing three participant groups using interview guides (Appendices 1–3).

### Analysis and integration

***Fidelity analysis.*** To quantify the level of agreement between the conduct of the RESSA and the protocol, we will calculate the percentage agreement. The percentage agreement will be determined using the following formula:


Percentageagreement=(Numberofagreed"metitems"/Totalnumberofitems)×100

Here, the Number of agreed "met items" refers to the number of checklist items where both researchers independently scored the item as "met". "Total Number of Items" refers to the total number of checklist items that have been assessed. This percentage agreement will be reported to indicate the level of consistency between the conduct of the RESSA and the protocol. A high percentage agreement will suggest strong fidelity to the RESSA protocol, while a lower percentage may indicate areas where the conduct diverged from the intended protocol. Additionally, the nature and reasons for any discrepancies will be documented and discussed to provide further insights into the fidelity assessment process.

Quantitative data will be analysed using descriptive statistics. Frequencies, means, and standard deviations will be calculated to summarise the number of key informants who declined, delegated, did not attend interviews, did not respond to the interview request, or refused to answer one or more questions.

The interview with the RESSA researcher will undergo thematic analysis, guided by a coding framework derived from the interview guide. This structured approach will ensure that the analysis remains aligned with the study’s objectives and key areas of inquiry. During the analysis, inductive coding will also be applied to identify themes that extend beyond the predefined framework, capturing unanticipated insights. Coding will be facilitated using NVivo V20 ( QSR International, 2020).

***Acceptability analysis.*** Interviews will be analysed using a deductive thematic approach, applying the seven constructs of the Theoretical Framework of Acceptability (affective attitude, burden, ethicality, coherence, opportunity costs, perceived effectiveness, and self-efficacy) as an initial coding framework. The coding framework will be refined iteratively as transcripts are reviewed. Inductive codes will be added to capture unanticipated themes. NVivo V20 will be used to manage, organise, and facilitate coding and analysis of qualitative data.

***Stakeholder experience analysis.*** Thematic analysis will be employed to explore stakeholder experiences, guided by a coding framework developed to reflect the study’s aims. The initial set of deductive codes will be informed by the interview guide, ensuring that the analysis remains focused on the study’s objectives. Inductive coding will be applied to capture unexpected and emergent themes. NVivo V20 will be used to manage and analyse the data to maintain consistency and credibility.

***Integration of findings.*** Findings from fidelity, acceptability, and stakeholder experience analyses will be synthesised to provide a comprehensive understanding of the implementation of the RESSA. Qualitative and quantitative findings will be triangulated to identify similarities and differences and to generate actionable recommendations for future RESSAs.

### Trustworthiness

Trustworthiness has been a consistent concern with qualitative research ( Nowell *et al.*, 2017). This protocol has made several provisions to address Guba’s criteria for trustworthiness ( Guba, 1981) in qualitative research to validate the findings.

We will adopt appropriate, well-recognised research methods, utilise random sampling of individuals serving as informants and utilise triangulation via the use of different methods and different types of informants. We will use iterative questioning in data collection dialogues and allow for peer scrutiny of the project. We will also provide an in-depth methodological description with detailed records of data collection and analysis procedures to provide an audit trail and allow for the study to be repeated, and also to allow the integrity of the results to be scrutinised.

### Ethical considerations

An application for the RESSA process evaluation was approved by the University of Galway Research Ethics Committee (Ref: 2024.01.003, Amend 2024.03, approval date 26-03-2024).

Participant information sheets and consent forms were approved during the ethics approval process. Written informed consent will be obtained from all participants before data collection. Participants will be provided with detailed information about the study, including its purpose, procedures, potential risks, and benefits. Confidentiality will be ensured by assigning unique identifiers to participants and securely storing consent forms. The study will be conducted in accordance with the Declaration of Helsinki.

### Data management and privacy

All audio recordings from interviews will be deleted after transcription. Only anonymised transcripts will be retained. These transcripts, along with any field notes and related documents, will be stored for a minimum of seven years in accordance with University of Galway policy.

In compliance with GDPR 2018 and the University of Galway Personal Data Security Schedule (PDSS), electronic records will be stored on the University of Galway OneDrive server. Access to these records will be through password-protected, encrypted laptops or desktops belonging to the research team. Consent forms will be securely stored either electronically in OneDrive or physically in a locked filing cabinet at the University of Galway. Individual names will not be linked to responses at any stage of the study.

Access to the data is restricted to the research team members: Dr. Marie Tierney, Professor Declan Devane, Dr. Nikita Burke, Dr. Paula Byrne and Dr. Barbara Whelan. If a professional transcription service is utilised, it will be bound by stringent data confidentiality agreements.

### Dissemination

The dissemination of the work of this process evaluation will be multi-faceted.

As previously noted, there has not been a framework for the conduct of a process evaluation of an assessment like a RESSA previously, so this protocol will serve to guide future process evaluations of RESSAs.

We will also publish the findings of the RESSA process evaluation and produce a report for our funders, the HRB. We anticipate that the findings of the RESSA process evaluation will offer insights into its effectiveness and help inform the methodology that could be applied across governmental departments, ultimately enhancing evidence-informed policymaking across the board.

Furthermore, we will present both the protocol and the findings of the process evaluation at the “RESSA Country Leads” meetings led by the Global Evidence Commission to provide insights to others working in this space internationally.

## Conclusion

This protocol outlines a structured evaluation of the Rapid Evidence Support System Assessment (RESSA) within Ireland’s Department of Health. By focusing on fidelity, acceptability, and stakeholder experiences, this study aims to enhance the relevance and impact of the RESSA methodology. The findings will contribute to refining how evidence support systems operate, improving their adaptability and robustness in diverse policy contexts.

As one of the first systematic evaluations of the RESSA, this study has the potential to inform evidence-informed policymaking both in Ireland and internationally. The results will offer insights into effective approaches for integrating evidence into policy development and decision-making processes, fostering a more consistent and context-sensitive application of evidence-informed policy. Ultimately, this protocol seeks to support the evolution of evidence support systems, enhancing their capacity to guide strategic decisions in government and beyond.

## Data Availability

No data are associated with this article. Open Science Framework: Process Evaluation of a Rapid Evidence Support System Assessment of Ireland’s Department of Health – A Protocol, https://doi.org/10.17605/OSF.IO/W5QAN ( Tierney, 2024). The extended data in this project include interview guides and data collection tools to measure the constructs of fidelity, acceptability and experiences among the three participant groups. It also pertains to the COREQ checklist. This project contains the following referenced extended data: Appendix 1: RESSA researcher interview guide Appendix 2: RESSA key informants interview guide Appendix 3: RESSA oversight group interview guide Appendix 4: COREQ checklist Data are available under the terms of the Creative Commons Attribution 4.0 International license (CC-BY 4.0). We will use the COnsolidated criteria for REporting Qualitative research (COREQ) checklist (Appendix 4), as developed by Tong *et al.* in 2007, when reporting our findings. The use of this checklist promotes explicit and comprehensive reporting of qualitative research and ensures that researchers provide sufficient detail on their methods of data analysis and the relationship between the analysis and the findings in the research report so that reviewers can assess the rigour of the analysis and the credibility of the findings.
