# Supplementary material for: Process Evaluation of a Rapid Evidence Support System Assessment of Ireland’s Department of Health – A Protocol
Source: HRB Open Res. 2025 Oct 13;8:22. Originally published 2025 Feb 3. [Version 2] doi: 10.12688/hrbopenres.14062.2 (PMC12576317; doi:10.12688/hrbopenres.14062.2)
Supplement: Peer review history

# Reviewer report: Reviewer response for version 2

**DOI:** 10.21956/hrbopenres.15694.r50729

**Published:** 2025-10-30

**Authors:** Annette Boaz

**Affiliations:** 1 King's College London, London, England, UK

The authors have now made it much clearer what the objectives of this paper are and how they relate to the protocol which was my main concern. I am happy to amend the recommendation to approve the manuscript.

Is the study design appropriate for the research question?

Yes

Is the rationale for, and objectives of, the study clearly described?

Partly

Are sufficient details of the methods provided to allow replication by others?

Yes

Are the datasets clearly presented in a useable and accessible format?

Yes

Reviewer Expertise:

NA

I confirm that I have read this submission and believe that I have an appropriate level of expertise to confirm that it is of an acceptable scientific standard.

# Reviewer report: Reviewer response for version 2

**DOI:** 10.21956/hrbopenres.15694.r50730

**Published:** 2025-10-22

**Authors:** Ellen Goldstein

**Affiliations:** 1 University of Illinois, Chicago, USA

In this paper, the authors seek to conduct a comprehensive process evaluation of the Rapid Evidence Support System Assessment (RESSA). The authors have incorporated the reviewers’ feedback to improve upon the quality and readability of this paper in clarity, specificity and organization. Findings from this research have the potential to inform methodology evaluating the effectiveness of system improvements and, as a result, enhance evidence-based  decision- and policymaking across institutions.

Is the study design appropriate for the research question?

Yes

Is the rationale for, and objectives of, the study clearly described?

Yes

Are sufficient details of the methods provided to allow replication by others?

Partly

Are the datasets clearly presented in a useable and accessible format?

Not applicable

Reviewer Expertise:

My areas of expertise are perinatal mental health; trauma-informed care; health system transformation and integration.

I confirm that I have read this submission and believe that I have an appropriate level of expertise to confirm that it is of an acceptable scientific standard.

# Reviewer report: Reviewer response for version 1

**DOI:** 10.21956/hrbopenres.15443.r48267

**Published:** 2025-08-14

**Authors:** Ellen Goldstein

**Affiliations:** 1 University of Illinois, Chicago, USA

Overall, the paper is well written and informative, however there are some structural and organizational changes that could improve upon its readability. For starters, substantive descriptions of the RESSA process belong in the methods section following the stated study aims. For example, the entire following paragraph describes methods, “In this RESSA, a flexible, mixed methods approach, integrating qualitative and quantitative analyses will be adopted…” The introduction would benefit from adhering to an outline: paragraph 1: establishing the broader context and significance; paragraph 2: narrowing the focus and reviewing relevant literature; paragraph 3: identifying the gap that your study seeks to fill; 4: stating the objectives/goal and aims of your study.

 The methods section necessitates extensive reorganization and omission of redundancies. For example, the paragraph describing the qualitative methods for conducting semi-structured interviews with stakeholders is repeated three different times in the methods section. The authors have already developed key categories for assessing fidelity, acceptability, and stakeholder experiences. Use these headings iteratively to describe the procedures, measures, and analysis required for each category.

 Explicate the following in the methods for better clarity: What is the anticipated/intended sample size of each representative participant group? Clearly describe your plan for recruitment per group. It is unclear how acceptability of intervention deliverers will be assessed? Consolidate the two separate sections on ethical considerations into one section. Informed consent is a study procedure that can be included under the heading of data management and privacy.

 A more complete, detailed, and systematic description of the 6-steps of the RESSA approach with a figure accompaniment would benefit this research as well as the intended audience. See citation below of RESSA pdf.

Is the study design appropriate for the research question?

Yes

Is the rationale for, and objectives of, the study clearly described?

Yes

Are sufficient details of the methods provided to allow replication by others?

Partly

Are the datasets clearly presented in a useable and accessible format?

Not applicable

Reviewer Expertise:

My areas of expertise are perinatal mental health; trauma-informed care; health system transformation and integration.

I confirm that I have read this submission and believe that I have an appropriate level of expertise to confirm that it is of an acceptable scientific standard, however I have significant reservations, as outlined above.

# Reviewer report: Reviewer response for version 1

**DOI:** 10.21956/hrbopenres.15443.r45366

**Published:** 2025-02-26

**Authors:** Annette Boaz, Mary Baginsky, Nicole Steils

**Affiliations:** 1 King's College London, London, England, UK

This paper has a tough communication task - describing plans for an evaluation of an evaluation tool evaluating an evidence support system. I read the protocol a couple of times trying to be clear what was being evaluated by the RESSA. I wondered if it would help to lift one of the elements out of the text and into a box to help the reader. This might be the description of the RESSA approach or of what is being evaluated in this case. 

 It would be useful to add in the introduction (maybe paragraph 4) that the RESSA is accompanied by a process evaluation. This would then be followed by introduction of the RESSA in the next paragraphs and then the process evaluation itself.

 It wasn't quite clear what was involved in the process evaluation and what was the RESSA itself. If anything can be done to make this clearer to the reader it would be a big help.

 In terms of stakeholders, is there an option to go broader and also engage with other government departments, e.g. education. If not, maybe highlight as a limitation as we know other government departments are addressing health challenges (mental health in schools, prisons etc.). Other Departments are involved in the oversight group so this is likely to come up in the process of conducting the evaluation.

 There are two sections on ethics and consent:  The final section and a sub-section on Ethical considerations. These sections contain slightly different content, but also some repetition so could be combined.

Is the study design appropriate for the research question?

Yes

Is the rationale for, and objectives of, the study clearly described?

Partly

Are sufficient details of the methods provided to allow replication by others?

Yes

Are the datasets clearly presented in a useable and accessible format?

Yes

Reviewer Expertise:

evidence use/ knowledge mobilisation

We confirm that we have read this submission and believe that we have an appropriate level of expertise to confirm that it is of an acceptable scientific standard, however we have significant reservations, as outlined above.
